# Inverse design of periodic microstructures with targeted nonlinear mechanical behaviour

**DOI:** 10.1007/s00158-024-03761-7

**Published:** 2024-03-18

**Authors:** Dilaksan Thillaithevan, Ryan Murphy, Robert Hewson, Matthew Santer

**Affiliations:** Department of Aeronautics, Imperial College London, London, UK

**Keywords:** Inverse homogenization, Inverse design, Topology optimization, Hyperelasticity, Finite strain, Periodic microstructures, Metamaterials

## Abstract

This paper introduces an inverse design framework for the precise tailoring of desired nonlinear mechanical responses in periodic microstructures, with particular focus on prescribed nonlinear stress–strain relationships. The topology optimization hinges on minimizing the error between the target and realized properties of the microstructures. A deformation-driven homogenization framework is setup. The periodic constraints needed for the microscale equilibrium equation are imposed through strongly enforced periodic boundary conditions and the removal of the translational nullspace, avoiding the need for Lagrange multipliers, greatly simplifying the implementation. Automatic differentiation is leveraged to efficiently calculate the necessary sensitivities for the gradient-based optimization. To further aid the design of discrete designs a intermediate density penalty constraint is proposed. Numerical examples underscore the efficacy of our methodology, showcasing microstructures that demonstrate targeted softening and stiffening as well as distinctive directional behaviour.

## Introduction

Structural metamaterials derive their mechanical properties from the distribution of material on the microscale, typically in the form of discrete microstructure geometries, rather than the properties given by the bulk material. Interest in structural metamaterials has greatly accelerated with the increased adoption of additive manufacturing (AM) technologies. Metamaterials offer the possibility of designing materials with highly tailored, application specific properties. One method for systematically designing such materials that has proven particularly successful is the use of topology optimization (TO) with solid isotropic material with penalization (SIMP) Bendsøe and Sigmund (1999) to tackle inverse design problems. Here the goal is to find the microscale material distribution which leads to the desired homogenized material properties, for example negative Poisson’s ratio (NPR) (Sigmund 1994; Andreassen et al. 2014) or negative thermal expansion designs (Sigmund and Torquato 1997). The design of NPR or auextic metamaterials, which transversally expand/contract when exposed to an axial stretch/compression, has been extensively studied. For example, Sigmund demonstrated the design of auxetic materials using a truss-based inverse design formulation Sigmund (1994). SIMP-based formulations were later used to design NPR designs Wang et al. (2014). SIMP-based TO has also been used to address thermal designs. For example in Sigmund and Torquato (1997) a three-phase setup (two material and void phase) was utilised to generate structures with negative thermal expansions.

While most inverse design frameworks assume linear properties, more recently, efforts have shifted towards the design of microstructures considering nonlinear effects. Experiments have shown that metamaterials can be highly sensitive to nonlinear deformations, for example in Choi and Lakes (1992) the authors showed that the level of auxeticity of NPR metamaterials is strain-dependent. To ensure designs are applicable in finite strain regimes and to better exploit the benefits of metamaterials, finite strain frameworks are needed. For example Wang et al. (2014) optimized microstructures under finite strains in a tensile test setting (i.e. by assuming a uniaxial stress state). It was shown that 2D auxetic designs optimized under the assumption of linear elasticity had strain-dependent auxetic properties and presented examples of auxetic microstructures designed under finite deformations to overcome this issue. This work was later extended to the design of 3D auxetic microstructures in Wang (2018). To avoid the formation of thin material regions, the authors utilised a three-phase projection formulation where, alongside the “blueprint” design, slightly eroded and dilated designs are considered in the optimization (Wang et al. 2011). This also provides some degree of robustness against manufacturing errors. Other examples of systematically designed nonlinear auxetic metamaterials have been presented in Wallin and Tortorelli (2020); Zhang and Khandelwal (2019). Wallin *et al. * imposed symmetry boundary conditions on the domain to design microstructures with orthotropic material properties, avoiding the formation of shear stresses and Zhang *et al. * presented microstructure designs optimized using different unit cell boundary shapes.

Systematic design of metamaterials with prescribed, nonlinear, constitutive material properties have also been demonstrated. For example, Behrou et al. (2021) showcased metamaterials designed to exhibit targeted stiffness tensors at given finite strain intervals. In Ivarsson et al. (2020) a framework for the design of visco-elastic metamaterials was presented. In this framework the optimization is setup to design materials with maximum viscoplastic energy absorption subject to displacement constraints. The authors also highlight the dependence of the final optimized geometries on the initial material distribution. This is a widely reported feature of periodic inverse design problems and is further explored in Section. 5.5.

Li et al. presented a formulation for programming the displacement and force maps for non-periodic (macroscale) structures Li et al. (2023). The authors were able to demonstrate the tailoring of structural responses, such as counter rotations and directional expansions and contractions. The goal of the optimization utilised in their work is in a similar vein as the one presented in this paper. On the other hand, in this paper we address the optimization of periodic microstructures, using nonlinear homogenization theory, with targeted stress–strain relationships using an inverse homogenization approach As a result, the methods and implementation presented in this differ significantly from the framework presented in Li et al. (2023). Programming the stress–strain relationships allows for the tailoring of nonlinear microstructure behaviour and generation of designs which exhibit different responses in various strain regimes. Targeted stress–strain relationships have been tackled in a truss-based framework in Wang et al. (2014) and very recently in Dalklint et al. (2023), where the authors utilise a third medium approach for modelling self-contact within the inverse homogenization procedure. Two main contributions are made to the state-of-art in this field:

1. A framework for systematically designing periodic microstructures with targeted nonlinear stress–strain relationships is developed and its effectiveness is demonstrated through several numerical optimization examples
2. An intermediate density penalty constraint formulation is presented to encourage the formation of discrete microstructure designs and further aid the optimization convergence.

This paper builds on the existing literature in inverse design methodologies by presenting a formulation for systematically designing metamaterials with prescribed nonlinear stress–strain responses. Using a materially linear but geometrically nonlinear hyperelastic model, a deformation-driven homogenization approach is utilised to derive the homogenized quantities required for the optimization. This paper is organized as follows. Section 2 presents the nonlinear, deformation-driven homogenization formulation and the finite element implementation. The material interpolation utilised is outlined in Section. 3. The topology optimization formation is presented in Section. 4, including the IDP constraint approach. The utility of the framework is validated through several numerical examples in Section. 5, including a study on the effect of different material distribution initializations. Finally, Section. 6 summarizes the main conclusions from this study.

## Deformation-driven nonlinear homogenization

Consider a multiscale structure, composed of periodic microstructures on the microscale, assembled to form a macroscale structure. On the microscale, a single periodic unit cell is defined on a domain composed of solid, ${\mathcal {S}}$, and void constituents, ${\mathcal {V}}$, with the cell domain defined as $\Omega _{m} = {\mathcal {S}} \cup {\mathcal {V}}$. The mixture of ${\mathcal {V}}$ and ${\mathcal {S}}$ introduces local, microscale, behaviour and so targeted structural behaviour can be achieved by optimizing the placement of material within the microscale domain. This process typically relies on a homogenization method to determine the homogenized microstructural properties.

The Method of Multiscale Virtual Power (MMVP) De Souza Neto et al. (2015) is used to perform homogenization. In general, homogenization hinges on two fundamental assumptions: first, a scale of separation, and second, periodicity of small scale fluctuations. Physically, the requirement for scale of separation requires the dimensions of the microscale unit cells to be much smaller than the macroscale. It should be noted the exact magnitude of either lengthscale is not of importance, their relative scale is the critical component. As a result of this scale of separation, microscale field variables fluctuate much faster than the macroscopic variables, which can be assumed to be uniform across a single unit cell.

### Linking nonlinear micro- and macroscale deformations

**Fig. 1:**
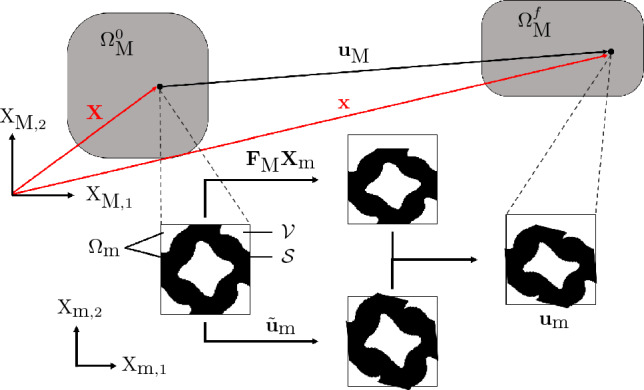
Illustration highlighting the interaction between the macro- and microscale deformation

When the multiscale structure is subjected to loading and displacements, the macroscale domain deforms from its initial configuration, $\Omega _\text {M}^0$ to a final configuration $\Omega _\text {M}^f$ via a nonlinear mapping, as shown in Fig. 1. Taking $\textbf{X}_\text {M} \in \Omega _\text {M}^0$ and $\textbf{x}_\text {M} \in \Omega _\text {M}^f$ as the points in the initial and final configurations, respectively, their locations are related by

1 $$\begin{aligned} \textbf{x}_\text {M} = \textbf{X}_\text {M} + \textbf{u}_\text {M} \end{aligned}$$

where $\textbf{u}_\text {M}$ is the displacement. Concurrently, a unit cell or representative volume element (RVE), located at point $\textbf{X}_\text {M}$ deforms from $\Omega _\text {m}^0$ to $\Omega _\text {m}^f$, where it should be noted that for the remainder of this paper subscripts $[\cdot ]_\text {m}$ and $[\cdot ]_\text {M}$ will be used to denote microscopic and macroscopic quantities, respectively. The updated microscale coordinate is determined using

2 $$\begin{aligned} \begin{aligned} \textbf{x}_\text {m} = {\textbf{F}}_\text {M}{\textbf{X}}_\text {m} + \tilde{\textbf{u}}_\text {m} \end{aligned} \end{aligned}$$

where $\tilde{\textbf{u}}_\text {m}$ is the microscale fluctuation, caused by the presence of the mixture of ${\mathcal {V}}$ and ${\mathcal {S}}$ in $\Omega _{\text {m}}$, and ${\textbf{F}}_\text {M}$ is the macroscale deformation gradient, defined as

3 $$\begin{aligned} {\textbf{F}}_\text {M} = \frac{\partial \textbf{x}_\text {M}}{\partial \textbf{X}_\text {M}} = \textbf{I} + \frac{\partial \textbf{u}_\text {M}}{\partial \textbf{X}_\text {M}} \end{aligned}$$

where $\textbf{I}$ is the identity operator. Using Eq. 2, the microscale displacement can be derived as

4 $$\begin{aligned} \begin{aligned} \textbf{u}_\text {m} = \textbf{u}_\text {M} + \tilde{\textbf{u}}_\text {m} \end{aligned} \end{aligned}$$

where it is evident that the total microscale displacement is composed of the macroscale, “mean”, displacement, $\textbf{u}_\text {M}$, and the microscale fluctuations, $\tilde{\textbf{u}}_\text {m}$.

Homogenization theory links the macro- and microscale deformation gradients and displacements through the volume averaging relationships

5 $$\begin{aligned} \begin{aligned} \textbf{F}_\text {M}&= \frac{1}{|\Omega _{\text {m}}|} \int _{\Omega _{\text {m}}} {\textbf{F}}_\text {m} \; d\Omega _{\text {m}} \\ \textbf{u}_\text {M}&= \frac{1}{|\Omega _{\text {m}}|} \int _{\Omega _{\text {m}}} {\textbf{u}}_\text {m} \; d\Omega _{\text {m}} \end{aligned} \end{aligned}$$

Using Eq. 2 and assuming $\int _{\Omega _{\text {m}}} \textbf{X}_\text {m} \; dV = 0$ and that ${\textbf{F}}_\text {M}$ is constant across the unit cell (due to the scale of separation), we arrive at the relation

6 $$\begin{aligned} \begin{aligned} {\textbf{F}}_\text {M} = \frac{1}{|\Omega _{\text {m}}|} \int _{\Omega _{\text {m}}} {\textbf{F}}_\text {m} \; d\Omega _{\text {m}} + \frac{1}{|\Omega _{\text {m}}|} \int _{\Omega _{\text {m}}} \frac{\partial \tilde{\textbf{u}}_\text {m}}{\partial \textbf{X}_\text {m}} \; d\Omega _{\text {m}} \end{aligned} \end{aligned}$$

Combining Eqs. 5 and 6 and applying the divergence theorem we are left with

7 $$\begin{aligned} \int _{\partial \Omega _{\text {m}}} \tilde{\textbf{u}}_\text {m} \otimes \textbf{n} \; d\text {S} = 0 \end{aligned}$$

where $\partial \Omega _{\text {m}}$ is the boundary of the unit cell and $\textbf{n}$ is the unit normal vector on $\partial \Omega _{\text {m}}$. Additionally, from Eq. 4 and the displacement homogenization relation in Eq. 5, it can be shown that

8 $$\begin{aligned} \int _{\Omega _{\text {m}}} \tilde{\textbf{u}}_\text {m} \; d\Omega _{\text {m}} = 0 \end{aligned}$$

These equations constrain $\tilde{\textbf{u}}_\text {m}$ to the space of kinematically admissible microscale fluctuations, ${\mathcal {H}}_\text {m}$,

9 $$\begin{aligned} \begin{aligned} {\mathcal {H}}_\text {m} = \Bigg \{ \tilde{\textbf{u}}_\text {m}: \tilde{\textbf{u}}_\text {m} \in {\mathcal {H}}^1,&\; \int _{\Omega _{\text {m}}} \tilde{\textbf{u}}_\text {m} \; d\Omega _{\text {m}} = 0, \\&\int _{\partial \Omega _{\text {m}}} \tilde{\textbf{u}}_\text {m} \otimes \textbf{n} \; d\text {S} = 0 \Bigg \} \end{aligned} \end{aligned}$$

where ${\mathcal {H}}^1$ is the space of quadratically integrable functions. In summary, Eqs. 7 and 8 constrain $\tilde{\textbf{u}}_\text {m}$ to the space of kinematically admissable displacements, ${\mathcal {H}}_\text {m}$, where we note that Eq. 7 removes rigid body rotations and Eq. 8 eliminates rigid body translations.

### Macro- microscale equilibrium

At a point, $\textbf{X}_\text {M} \in \Omega _M$, in the absence of external forces, using the Hill-Mandel Principle, the virtual power equilibrium across the macro- and microscale can be defined as De Souza Neto et al. (2015)

10 $$\begin{aligned} {\mathcal {P}}_{\text {M},\textbf{X}} = {\mathcal {P}}_{\text {m}, \textbf{X}} \end{aligned}$$

where ${\mathcal {P}}_\text {M}$ and ${\mathcal {P}}_\text {m}$ are the virtual, internal macro- and microscale powers. Using the First Piola-Kirchhoff stress, $\textbf{P}$, macroscale power is defined as

11 $$\begin{aligned} {\mathcal {P}}_\text {M} = \textbf{P}_\text {M} \cdot \nabla _{\textbf{X}_\text {M}} {{\hat{\textbf{u}}}}_\text {M} \end{aligned}$$

where $\textbf{P}_\text {M}$ is the macroscale First Piola-Kirchhoff stress, not to be confused with the macroscale power, ${\mathcal {P}}_\text {M}$, and $\hat{[\cdot ]}$ is used to denote virtual quantities. Utilising the identity provided in Eq. 4, the microscale power can then be defined as a volume averaged quantity over the microscale domain

12 $$\begin{aligned} \begin{aligned} {\mathcal {P}}_\text {m} = \frac{1}{|\Omega _{\text {m}}|}\int _{\Omega _{\text {m}}}&\textbf{P}_\text {m} \cdot \bigg [\nabla _{\textbf{X}_\text {M}} {\hat{\textbf{u}}}_\text {M} + \nabla _{\textbf{X}_\text {m}} {\hat{{\tilde{\textbf{u}}}}}_\text {m} \bigg ] \; d\Omega _{\text {m}}, \\&\forall \; {\hat{{\tilde{\textbf{u}}}}}_\text {m} \in {\mathcal {H}}_\text {m} \end{aligned} \end{aligned}$$

Finally, by choosing $\nabla _{\textbf{X}_\text {M}} {\hat{\textbf{u}}}_\text {M} =0$ and consolidating the macro- and microscale equations above leads to the equilibrium equation

13 $$\begin{aligned} \begin{aligned} 0 = \int _{\Omega _{\text {m}}}&\textbf{P}_\text {m} \cdot \nabla _{\textbf{X}_\text {m}} {\hat{{\tilde{\textbf{u}}}}}_\text {m} \; d\Omega _{\text {m}}, \; \forall \; {\hat{{\tilde{\textbf{u}}}}}_\text {m} \in {\mathcal {H}}_\text {m} \end{aligned} \end{aligned}$$

### Finite element formulation

In this work the open-source finite element (FE) library, Firedrake Ham et al. (2023), is used to solve the periodic microscale fluctuation problem described in Eq. 13. Firedrake provides a simple solution for strongly enforcing periodic boundary conditions (PBCs) and evaluating objective and constraint sensitivities to the design variables using automatic differentiation (AD). Discussions surrounding the implementation and efficiency of AD within Firedrake are outside the scope of this work, the reader is referred to Farrell et al. (2012) for further details.

#### Periodic boundary conditions and rigid body translations

To facilitate the use of homogenization theory, the microscale problem is assumed to be periodic by imposing (PBCs) on the microscale domain. PBCs are imposed by setting displacements on opposing faces of a square domain to be equal, i.e. $\textbf{u}^+_\text {m} = \textbf{u}^-_\text {m}$ on $\partial \Omega _{\text {m}}$. This condition naturally satisfies the second kinematic admissibility constraint of zero microscale boundary fluctuations, as shown in Eq. 7. The remaining condition stipulating null average microscale fluctuations can be enforced by fixing an arbitrary point on the microscale, e.g. $\textbf{u}_{\text {m},i} =0 \in \Omega _{\text {m}} \; \forall \, i$.

In inverse design literature, the problem defined by Eqs. 13 and 9 are typically solved using a Lagrange multiplier approach to enforce $\textbf{u}^+_\text {m} = \textbf{u}^-_\text {m}$ and $\textbf{u}_{\text {m},i} =0$. This requires Eq. 13 to be modified to include the necessary Lagrange multipliers which enforce the aforementioned microscale displacement constraints. In this work an alternative, simpler, approach is utilised. The PBCs are handled automatically during the construction of the finite element mesh in Firedrake. The meshing process begins by creating a torus mesh which is subsequently mapped onto unit square domain boundary. This ensures that every secondary (or dependent) boundary in the mesh is topologically analogous to its corresponding primary boundary on the opposing face. Further, the degrees of freedom associated with these secondary boundaries are identified as those on the primary boundaries, thereby strongly enforcing PBCs on the domain.

Finally, as an alternative to fixing a single point to impose null average microscopic fluctuations, as shown in Eq. 8, the (null) space of rigid body translations are instead removed from the solution space prior to solving the nonlinear system of equations. Together, the strongly enforced PBCs and null space removal, provide a very simple path to solving the macro- microscale equilibrium problem.

### Homogenized stress

The goal of the optimization framework is to generate periodic microstructures with prescribed stress–strain relationships. Consequently, we are interested in obtaining the homogenized stress generated for any given material distribution in the microscale domain and resulting $\tilde{\textbf{u}}_\text {m}$. Noting a uniform $\nabla _{\textbf{X}_\text {M}} {\hat{\textbf{u}}}_\text {M}$ across the unit cell and taking $\nabla _{\textbf{X}_\text {m}} {\hat{\tilde{{\textbf{u}}}}}_\text {m} = 0$, the homogenized stress can be derived as

14 $$\begin{aligned} \begin{aligned} \textbf{P}_\text {M} = \frac{1}{|\Omega _{\text {m}}|}\int _{\Omega _{\text {m}}}\textbf{P}_\text {m} \; d\Omega _{\text {m}} \end{aligned} \end{aligned}$$

In this work a 2D plane strain model is used alongside the modified St. Venant-Kirchoff (SVK) hyperelastic model is used to describe the material behaviour Klarbring and Strömberg (2013). This is used to overcome the well documented limitation of the classical SVK hyper elastic model which exhibits artificial softening during compression. The modified SVK model has been used extensively in nonlinear TO literature, for example in Wang et al. (2014); Klarbring and Strömberg (2013). Furthermore, for the strain regime utilised in this work, the modified SVK model has been shown to exhibit near-identical behaviour to Neo-Hookean hyperelastic models Klarbring and Strömberg (2013); Wang et al. (2014). It should also be noted that alternative hyperelastic models (e.g. Neo-Hookean) could be used with very minor modifications to the framework. The modified SVK model used in this work is defined as

15 $$\begin{aligned} \begin{aligned} {\psi } = \frac{1}{2}\lambda (\text {J}-1)^2 + \mu \;\text {Tr}(\textbf{E}^2) \end{aligned} \end{aligned}$$

where the invariant $J = \text {Det}(\textbf{F})$. Lamé parameters are denoted by $\lambda$ and $\mu$ and $\textbf{E}$ is the Green-Lagrange strain tensor and is the source of geometric nonlinearity, defined as $\textbf{E} = \frac{1}{2}(\textbf{C} - \textbf{I})$ and $\textbf{C} = \textbf{F}^T\textbf{F}$ is the right Cauchy-Green tensor.

To summarise, for a given macrostrain, $\frac{\partial \textbf{u}_\text {M}}{\partial \textbf{X}_\text {M}}$, we solve Eq. 13 for $\tilde{\textbf{u}}_\text {m}$. The microscale stress, $\textbf{P}_\text {m}$ is then computed and volume-averaged to give $\textbf{P}_\text {M}$.

## Material interpolation

The interaction between the design variables (material distribution) and $\mathbf {{u}}_\text {M}$ is fundamentally driven by SIMP interpolation (Bendsøe and Sigmund 1999), where in a discretized domain, the elemental stiffness, $\text {E}_e$ is given by

16 $$\begin{aligned} \begin{aligned} \text {E}_e = {\rho }_e ^{\text {p}} (\text {E}_\text {s} - \text {E}_\text {v}) + \text {E}_\text {v} \end{aligned} \end{aligned}$$

where $\rho _e$ is the *e*-th elemental pseudo-density, $\text {E}_\text {s} = 1 \,$Pa and $\text {E}_\text {v} = 10^{-4 \;}$Pa are the solid and void material Young’s modulus, respectively, $p =3$ is the penalty parameter and $\nu$ is the Poisson’s ratio, set to 0.3 in this work. The SIMP penalised elemental stiffness then drives Eq. 15 through Lame’s paramters, given as

17 $$\begin{aligned} \begin{aligned} \lambda _e&= \frac{\text {E}_e\nu }{(1-\nu )(1-2\nu )} \\ \mu _e&= \frac{\text {E}_e}{2(1+\nu )} \end{aligned} \end{aligned}$$

To avoid checker-boarding, the material distribution is smoothed using a Helmholtz filtering scheme, where the design variables, $\rho$, are filtering by finding the Helmholtz-type PDE given by Lazarov and Sigmund (2011)

18 $$\begin{aligned} {\tilde{\rho }} - l^2 \nabla ^2{\tilde{\rho }} = \rho \end{aligned}$$

where ${\tilde{\rho }}$ are the smoothed design variables with length scale *l* (e.g. $l \rightarrow 0$, $\rho ={\tilde{\rho }}$). The filter length scale is set to $l = 5h$, where *h* is the cell width (i.e. $h=\frac{L}{n_e}$ where $L=1$ is the domain length and $n_e$ is the number of elements along *L*). It should be noted that the Helmholtz PDE shown above is discretized using the same periodic mesh used to discretize the hyperelastic equilibrium equation shown in Eq. 13. As a result, PBCs are strongly enforced on the Helmholtz PDE, as explained in Section. 2.3.1. A natural consequence of the Helmholtz smoothing scheme is the introduction of intermediate densities. To design purely “black” and “white” designs (i.e. containing only $\rho = 0 \vee \rho = 1$) projection filtering is typically applied to push the design variables towards 0 or 1, using the projection function Guest et al. (2004); Wang et al. (2011)

19 $$\begin{aligned} \begin{aligned} \bar{{\rho }} = \frac{\tanh {\big (\beta \eta \big )}+\tanh {\big (\beta ({{\tilde{\rho }}}-\eta )\big )}}{\tanh {\big (\beta \eta \big )}+\tanh {\big (\beta (1-\eta )\big )}} \end{aligned} \end{aligned}$$

where $\bar{{\rho }}$ are the projected design variables and $\eta$ are the threshold parameters, where ${\tilde{\rho }} > \eta$ are pushed towards 1 and ${\tilde{\rho }} < \eta$ are pushed towards 0, with $\eta = 0.5$. The linearity of the projection is determined by $\beta$, where as $\beta \rightarrow \infty$, the projection filter encourages binary designs. Large values of $\beta$ can lead to stability issues in the optimization. Consequently, $\beta$ is typically incremented during the optimization. However, similar to formulations targeting Poisson’s ratio objectives, the objective function (stress–strain relationship) utilised in this work does not naturally penalise grey material. As a result, an increase in $\beta$ alone does not encourage discrete designs. Modifications to the objective function and or additional constraints are needed to discourage grey material usage. To this end, we propose a grey material penalty function which encourages the optimizer towards discrete designs, as further explained in the subsequent section. When used in conjunction with a multi-objective formulation, where the stress errors and volume fraction are minimised, we are able to design discrete microstructure designs with crisp boundaries and low errors.

## Topology optimization

In this work we optimize the material distribution within a dimensionless, periodic, microscale unit cell ($\Omega _{\text {m}} = [0,1] \times [0,1]$) to derive desired material behaviour across a range of finite strain configurations. The objective function therefore is to minimize the error between the targeted and realised behaviour.

### Prescribed stress–strain relationship

Given *C* strain states (load cases), the objective function is defined as

20 $$\begin{aligned} \begin{aligned} {\min _\rho } \; {\mathcal {J}} = \max \Bigg [\Big |\textbf{P}_{\text {M},i}(\rho , {\tilde{\textbf{u}}}_\text {m}(\nabla {\textbf{u}}_{\text {M},i})) -&\textbf{P}_{\text {M},i}^*\Big |\Bigg ], \\ {}&\forall i \in \{1,\dots , \text {C}\} \end{aligned} \end{aligned}$$

where $\textbf{P}^*_i$ and $\textbf{P}_i$ are the target and realised stresses for *i*-th strain configurations, respectively, defined as $$\nabla {\textbf{u}}_{\text {M},i} = \begin{bmatrix} \varepsilon _{xx} &{} \varepsilon _{xy}\\ \varepsilon _{yx} &{} \varepsilon _{yy} \end{bmatrix}$$. The objective utilised in this work tailors the First Piola-Kirchhoff stress, this can easily be updated to tailor the Cauchy stress instead, depending on the needs of the designer. As the $\max$ function is discontinuous, a *p*-norm formulation is utilised instead. To this end, we reformulate the objective as

21 $$\begin{aligned} \begin{aligned} {\min _\rho } \; {\mathcal {J}}^{\text {PN}}&= \Bigg [\frac{1}{\text {C}}\sum _{i=1}^{C} e_i^p\Bigg ]^{1/p} \; \forall i \in \{1,\dots , \text {C}\} \\ e_i&= \frac{1}{\textbf{P}_{\text {M},i}^*} \Big (\textbf{P}_{\text {M},i}(\rho , {{\tilde{\textbf{u}}}}_\text {m}) - (\textbf{P}_{\text {M},i}\Big ) \end{aligned} \end{aligned}$$

where $p \in 2{\mathbb {N}}$ the controls the norm such that as $p \rightarrow \infty$, ${\mathcal {J}}^{\text {PN}} \rightarrow {\mathcal {J}}$, at the cost of increasing nonlinearity. Note that we normalise the error term, $\textbf{P}_{\text {M},i}(\rho , {{\tilde{\textbf{u}}}}_\text {m}) - \textbf{P}_{\text {M},i}^*$, using the target values, $\textbf{P}_{\text {M},i}^*$, to avoid bias toward larger target stress values. To impose a length scale on the designs, the three-phase projection method, Sigmund (2009); Clausen and Andreassen (2017) is utilised. During the optimization, alongside the base design ($\eta = 0.5$), two additional permutations of the design are evaluated with $\eta = \{0.55, 0.45\}$ to simulate eroded and dilated designs. The objective is then updated to minimise the errors across the three designs for each optimization iteration

22 $$\begin{aligned} {\min _\rho } \; {\mathcal {J}}^{\text {R}} = \Big [\frac{1}{3}\sum _{i} \big ({\mathcal {J}}^{\text {PN}}(\eta _i)\big )^{p_\text {R}}\Big ]^{1/p_\text {R}}, \; \eta _i \in \{\eta _{\text {e}}, \eta _{\text {b}}, \eta _{\text {d}}\} \end{aligned}$$

where the subscripts $\text {e}$, $\text {b}$ and $\text {d}$ indicate the eroded, base and dilated designs evaluated on the stress error function, ${\mathcal {J}}^{\text {PN}}$, with $\eta = \{0.55, 0.5, 0.45\}$, respectively and the norm is given by $p_\text {R}=2$, which was found to be sufficient for the examples presented in this work. Additional results are included in the Appendix to highlight the influence of the choice of norm. Using the length scale calculations provided in Wang et al. (2011), with a filter length, $l = 5h$ and $\eta = \{0.55,0.45\}$, a minimum length scale of 0.052 using is achieved. It should be noted that higher norms ($>2$) could be used in the robust formulation above to better capture the worst performing design, albeit at the cost of increased nonlinearity. For the problems tackled in this work the 2-norm is deemed sufficient since it results discrete structures with low errors while avoiding the formation of thin hinges. To avoid confusion, we only present the blueprint design ($\eta = 0.5$) in the subsequent sections.

### Intermediate density penalty

**Fig. 2:**
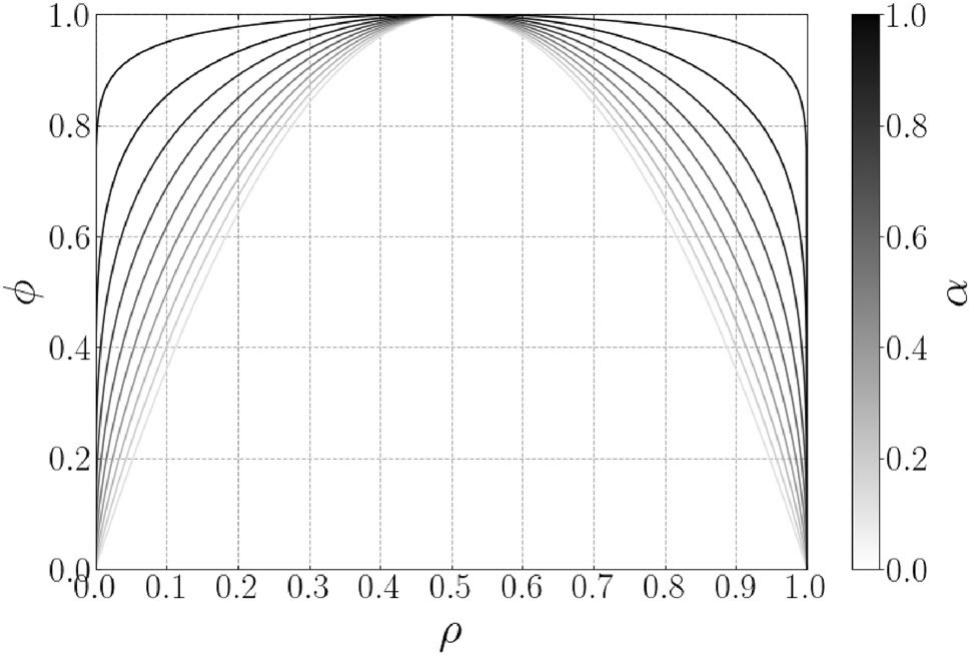
Relationship between intermediate density penalty function and design variables

Ideally, the optimized design variables should minimise the desired objective function through a binary, black/white material distribution (e.g. $\rho \approx 0$ $\vee$ $\rho \approx 1$ only). When material usage is restricted and given objectives and or constraints which penalise intermediate materials, for example by impose lower stiffness to weight ratios for intermediate materials, the optimizer is directed towards black and white designs as this is the most efficient use of material. As the objective (stress error), shown in Eq. 22 does not naturally penalise stiffness, imposing material usage constraints on their own would not encourage discrete designs. This is a known issue and problems of similar nature (for example Poisson’s ratio optimization) are typically tackled by imposing minimum stiffness constraints alongside a material usage constraint or by using a multi-objective formulation where a material usage term is added to the objective. The multi-objective formulation requires careful selection of scaling for the objective terms and a minimum stiffness constraint is not applicable for the objective considered in this work since the stiffness of the structure is implicitly specified through the stress–strain relationship (gradient). Instead, in this work we present the intermediate density penalty (IDP) constraint approach for generating black and white designs, inspired by the discreteness measure proposed in Sigmund (2007). In the absence of an objective function which penalises intermediate materials, this constraint directly penalises grey material in the design and is defined as

23 $$\begin{aligned} \phi = \frac{1}{N_e}\sum _{i=1}^{N_{e}}\Big [4 {\bar{\rho _i}} (1 - {\bar{\rho _i}})\Big ]^{(1 - \alpha )} \le \phi _{\max } \end{aligned}$$

where $\alpha \in [0,1)$ controls the nonlinearity of the penalty function, as shown in Fig. 2, and $\phi \in [0,1]$ is the intermediate density penalty, where $\phi = 0$ indicates a fully discrete design (${{\bar{\rho }}} = 0 \; \vee \; {{\bar{\rho }}} = 1$ only) and $\phi = 1$ represents a fully grey design with $\rho = 0.5$ everywhere. This constraint function bears similarities to the double-well potential functions that have been utilized in level-set and phase-field methods Jiang and Chen (2017); Yamasaki et al. (2015) and is in similar vein to the penalization constraint proposed in Borrvall and Petersson (2001). However, the parametric form with variable intermediate density penalization, shown in Eq. 23, has not previously been demonstrated. As $\alpha$ is increased the penalty for non-discrete designs increases as grey values closer to $\rho = 0$ and $\rho =1$ are more heavily penalised, as shown in Fig. 2. During the optimization we then control the discreteness of the design through the grey material upper bound, $\phi _{\max }$. To avoid restricting the optimizer at the beginning of the optimization, the optimization is initialized with a large value of $\phi _{\max }$ and small value of $\alpha$ which is updated through successive offline continuation steps. Further details of the update scheme will be provided in Section. 5. This constraint is utilised alongside material usage term in the objective to derive discrete designs.

To summarise, the target stress–strain inverse design problem is formulated as

24 $$\begin{aligned} \begin{aligned} {\min _\rho } \; {\mathcal {J}}^{\text {T}} = \;&{\mathcal {J}}^{\text {R}} + c V_f \\ \text {s.t. }: \;&0 \le \rho _{\text {min}} \le \rho \le \rho _{\text {max}} \\ 0 = \;&\textbf{r}({{\tilde{\textbf{u}}}}_{\text {m},i}(\rho )), \; \forall \; {{\tilde{\textbf{u}}}}_\text {m} \in {\mathcal {H}}_\text {m} \\ \phi = \;&\int _{\Omega _{\text {m}}}\Big [4 {{\bar{\rho }}} (1 - {{\bar{\rho }}})\Big ]^{(1 - \alpha )} d\Omega _{\text {m}}\le \phi _{\max }, \; \alpha \in [0, 1) \\ \end{aligned} \end{aligned}$$

where $V_f = \frac{1}{V}\int {\bar{\rho }} v \; d\Omega _{\text {m}}$ is the total material usage, *V* is the domain volume, *v* is the elemental volume and *c* is a scaling parameter. The lower and upper design variable bounds are given by $\rho _{\text {min}}$ and $\rho _{\text {max}}$, respectively, where $\rho _{\text {min}} =10^{-4}$ and $\textbf{r}$ is the residual of the nonlinear equilibrium equation, given in Eq. 13. It should be noted that due to the $E_\text {v}$ term in Eq. 16, the true void stiffness is $10^{-4}$ (for $\text {p}=3$) and as such $\rho _{\text {min}}$ is not strictly required. This relatively high void stiffness is employed (typically set to $E_{\text {v}} \le 10^{-6}\;$ Pa in literature) alongside the adapted Newton solver to help alleviate numerical instabilities caused by low density elements during the optimization, as outlined in Section. 5.

### Sensitivity analysis

As a gradient-based optimization algorithm is utilised, sensitives of the objective and constraint function are needed. The FE implementation in Firedrake provides a simple and efficient method for obtaining sensitivities through its automatic differentiation capabilities.

#### Objective sensitivities

The sensitives of the design variables, $\rho$, with respect to the objective function, shown in Eq. 24, are obtained through a mix of chain-rule and adjoint capabilities of Firedrake. The objective function sensitivity is given as

25 $$\begin{aligned} \frac{{\mathcal {J}}^{\text {T}}}{d\rho } = \frac{d {\mathcal {J}}^{\text {R}}}{d\rho } + c\frac{dV_f}{d\rho } \end{aligned}$$

where the material usage sensitivity, $\frac{d V_f}{d\rho }$ and the stress sensitivity, $\frac{d \textbf{P}_{\text {M},i}}{d{\rho }}$, are computed using automatic differentiation within Firedrake (the chain rule is used to compute the total derivative, $\frac{d{\mathcal {J}}^{\text {R}}}{d\rho }$).

### Material distribution initialization

Typically, in (linear) non-periodic structural optimization problems the design domain is initialised with a homogeneous material distribution. The introduction of boundary conditions results to a heterogeneous displacement field, which in turn provides heterogeneous sensitivities that a gradient-based optimizer is able to use to guide the optimization trajectory. While the designer selects the initial homogeneous material, the heterogeneity of the sensitivities is determined by the boundary conditions, limiting the influence (bias) of the designers’ initialisation. This is not the case in periodic, inverse design problems. Firstly, in a periodic setting, when employing homogenization approaches, it is the microscale material heterogeneity that leads to heterogeneous microscale displacements. A homogeneous microscale material distribution would result in a homogeneous, null, microscale displacement field and homogeneous sensitivities, restricting the optimization procedure. As a result, periodic inverse design problems are initialised with non-uniform material distributions, chosen by the designer. To this end, in this work we explore several initialisations to highlight the impact of the the initial material distribution on the optimized topologies and their performance.

### Optimization procedure

The gradient-based optimization algorithm, IPOPT (Wächter and Biegler 2006), is used to tackle the inverse design problems. Continuation schemes are utilised to update several parameters during the optimization avoid restricting the optimizer in the initial phases of the optimization, enabling the formation of discrete designs that sufficiently minimise errors. As IPOPT is a second-order method, it does not lend itself to online continuation, as is typically utilised in SIMP-based TO. Instead we employ an offline continuation approach where *K* successive sub-optimization problems are solved, each with updated parameters and using the *k*-1 solution to “warm-start” every *k*-th sub-optimization problem. Unless otherwise stated, all problems shown in this work are tackled using 6 continuation steps, that is by solving $K=6$ sub-optimization problems. The complete list of variables updated at each continuation step is shown in Table 1. The IDP constraint is used alongside SIMP (*p* = 3) to encourage the formation of discrete designs. From our testing, compared to un-penalized SIMP, it is found that the combination of IDP and SIMP penalization leads to designs which contain lower levels of grey material and have lower errors. Optimizing using the IDP constraint alone, leads to intermediate material being present for longer at the early stages of the optimization, which hinders the formation of nonlinear behaviour that is sought after (since grey material is not conducive to nonlinear behaviour), leading to errors over 10% at the end of the optimization. For comparison, designs optimized using unpenalized SIMP (*p* = 1) and the IDP constraint are given in Fig. 14. The projection filter, objective scaling, IDP parameter and limits are updated to encourage discrete designs and updates to the *p*-norm parameter increasingly targets the worst load-case. Following the first continuation the projection filter parameter, $\beta$ is updated using $\beta = 4k$ and the *p*-norm parameter is updated with $p = 2k$. The IDP parameter, $\alpha$ is linearly scaled between 0.05 and 0.95 over the 6 continuation steps to increase the penalty for grey materials in each continuation step. The constraint limit is incremented from $\phi ^0({\tilde{\rho }}) = 35\%$ to $\phi ^0({\tilde{\rho }}) = 7.5\%$ in the final continuation step.

**Table 1:** Parameters used in the topology optimization problem

Description	Symbol	Initial value	Maximum value
Continuation step	*k*	1	6
Projection curvature	$\beta$	2	20
*p*-norm	*p*	2	12
IDP parameter	$\alpha$	0.05	0.95
IDP limit	$\phi _{\max }$	$0.35 ^ {(1 - 0.05)}$	$0.075 ^ {(1 - 0.95)}$
Objective scaling	*c*	0	0.005

## Inverse design with targeted stress–strain relationships

The optimization formulation presented in Eq. 24 is utilised to solve a series of target stress–strain problems. In all examples a discretized mesh with 100 $\times$ 100, quad elements are utilised. Offline continuation is used to update the parameters shown in Table 1, with a total of 6 continuation steps. For avoidance of confusion, only baseline results ($\eta = 0.5$) are presented.

To demonstrate the utility of the inverse design framework we tackle four optimization problems; bi-directional softening in compression, directional softening in compression, bi-directional stiffening in tension and directional stiffening in tension. In each case, a series of macro-strains are imposed. For each strain configuration a target homogenized stress response is sought after. By specifying a series of macro-strains and target stresses we define a targeted, nonlinear, stress–strain relationship. In all cases only the direct strain terms are prescribed non-zero values, i.e. zero shear and transverse strains are applied.

The microscale problem, given by Eq. 13, is solved by using the Newton-Raphson method using the finite element framework provided by Firedrake. While it is not utilised in this work, energy interpolation can be included improve the convergence of the microscale problem Wang et al. (2014). Instead, the numerical instabilities arising due to the low density elements are tackled through a combination of a larger than usual void stiffness value and by using an adapted Newton solver. With regards to the void stiffness value, the responses of the optimized designs were validated using smaller void stiffness values which were found to have a negligible impact on the structural responses. Regarding the adapted Newton solver, we first attempt to solve the problem using the given strain configuration. If this fails to converge then we solve the problem for an intermediate strain point, halfway between the current target strain and the previously solved strain case. This is repeated until overall convergence is reached. Energy interpolation methods may be particularly important when considering larger finite strains. Furthermore, to consider more complicated nonlinear responses, arc length methods are required; this will be the focus of future research.

### Bi-directional softening

**Fig. 3:**
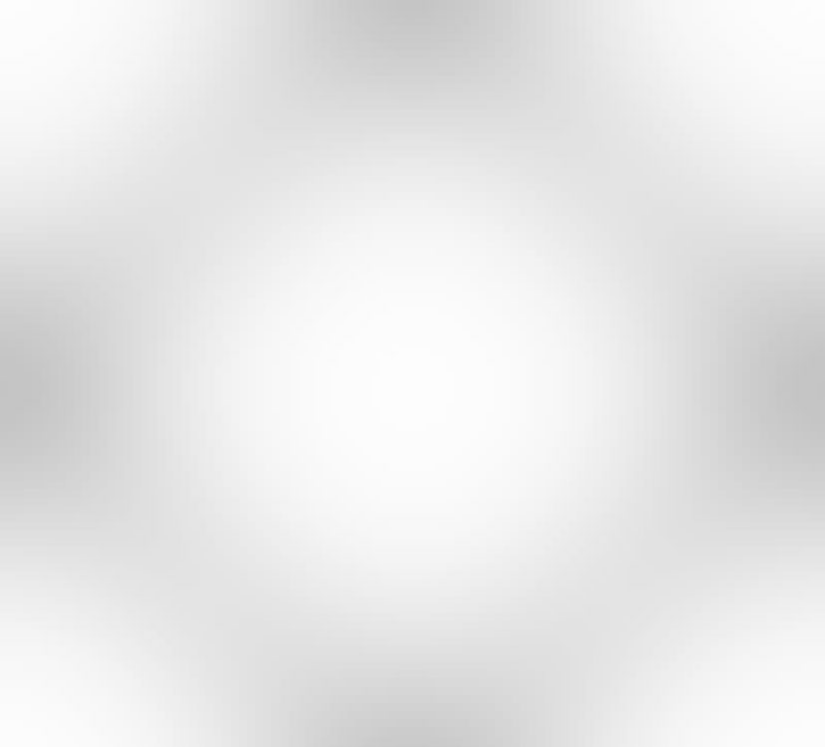
Smoothed diamond material distribution used to initialise inverse design problems

In this first example we seek designs with targeted bi-directional softening. The initial optimization ($k=1$) is initialised using a smoothed diamond material distribution, as shown in Fig. 3, with 150 optimization iterations for *k*=1 and 100 iterations in subsequent continuation steps. For the optimization we use three strain configurations, $\varepsilon _{xx}, \varepsilon _{yy} = [-0.05, -0.1, -0.15]$ to minimize the computational burden involved with considering additional strain configurations. The strain configurations are independently applied in each axial direction (e.g. the first load case in the *x*-direction is $$\begin{bmatrix} -0.05 &{} 0 \\ 0 &{} 0 \end{bmatrix}$$.

**Fig. 4:**
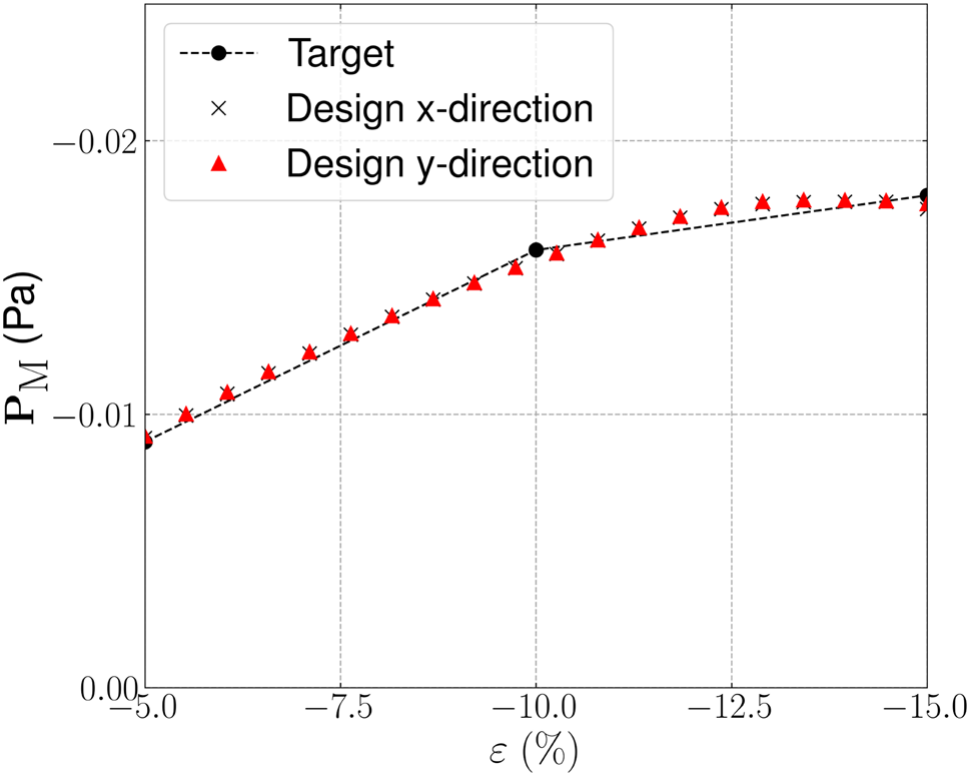
Bi-directional softening—target, initial and optimized finite deformation-based stress–strain relationships in the strain range $\varepsilon \in [-0.05, -0.15]$

**Fig. 5:**
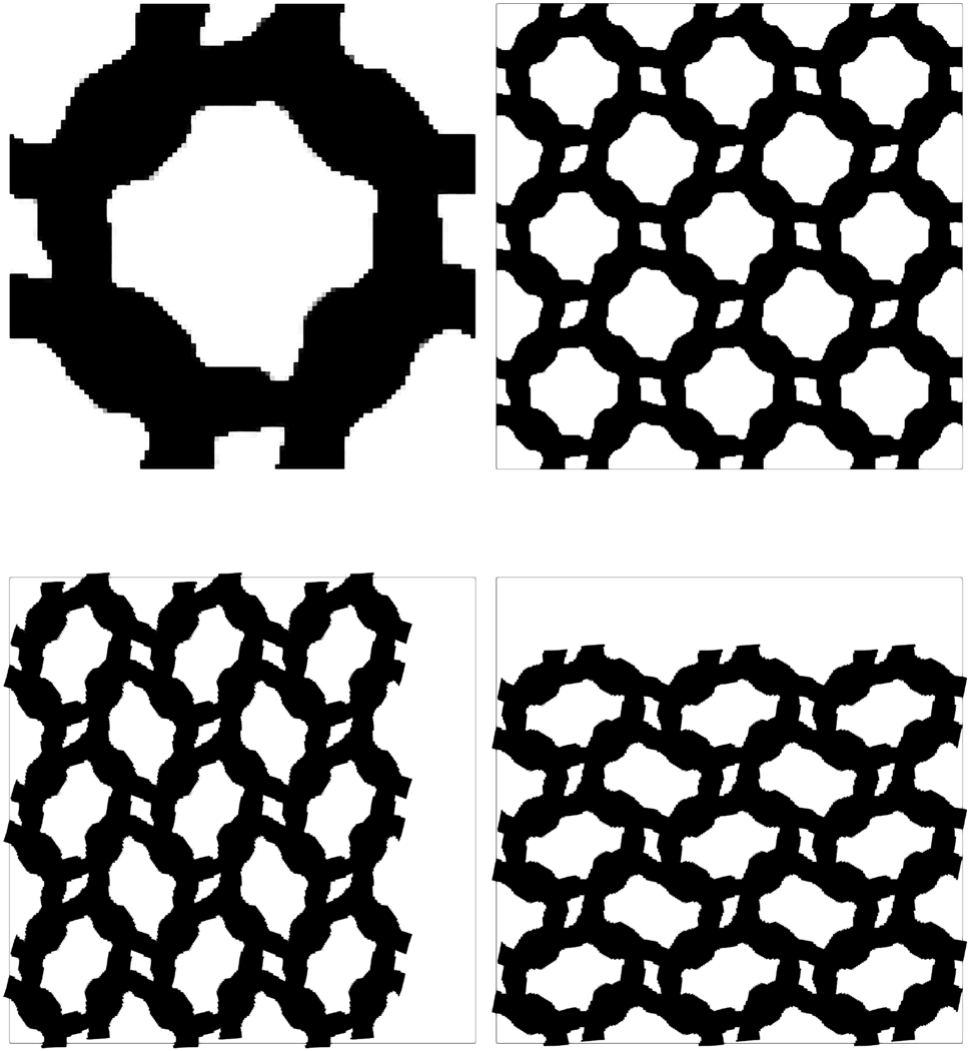
Geometry optimized for bi-directional softening. **a** Unit geometry (${\mathcal {J}}^{\text {PN}}_b = 0.026$, $\phi ^{0}=0.982\%$), **b** array of un-deformed 3$\times$3 unit cells, c) 3$\times$3 array deformed in x-direction ($\varepsilon _{xx} = -0.15$), d) 3$\times$3 array deformed in y-direction ($\varepsilon _{yy} = -0.15$). The black box indicates the outline of the un-deformed geometry

The target, initial and optimized stress–strain relationships are shown in Fig. 4. The target stress–strain relationship is defined at discrete strains (i.e. $\varepsilon =[-0.05, -0.1, -0.15]$), the dashed line connecting the individual target points has been included to more clearly highlight the change in gradient that is desired. The results for the optimized design are presented using 20 points in the strain range [$-$0.05, $-$0.15] to examine the behaviour at off-design points and to highlight that three strain design points are sufficient for the optimizer to replicate the targeted stress–strain relationship. The optimized stress–strain relationship is able to closely match the targeted relationship, with ${\mathcal {J}}^{\text {PN}}_b = 0.026$, displaying softening beyond 10% compressive strain in both axial directions and errors below 3% in all load cases. Similar performance is found for the eroded ($\eta = 0.53$) and dilated ($\eta = 0.47$) designs, with errors below 3%. The corresponding un-deformed and deformed geometries are shown in Fig. 5a. The geometry contains crisp boundaries, with $\phi ^{0} = 0.982\%$ indicating very low levels of grey material due to the addition of the IDP constraint. The deformed geometries are shown Fig. 5c and d. Here we observe the mesh like geometry is deforming in bending as it is compressed to 15%, causing the softening effect that we seek. The impact of the norm, $p_{\text {R}}$, used in the robust formulation, described in Eq. 22, is included in Appendix 2. It is found that increasing $p_{\text {R}}$ has a minimal on the material distribution but leads to larger errors compared to $p_{\text {R}}=2$.

**Fig. 6:**
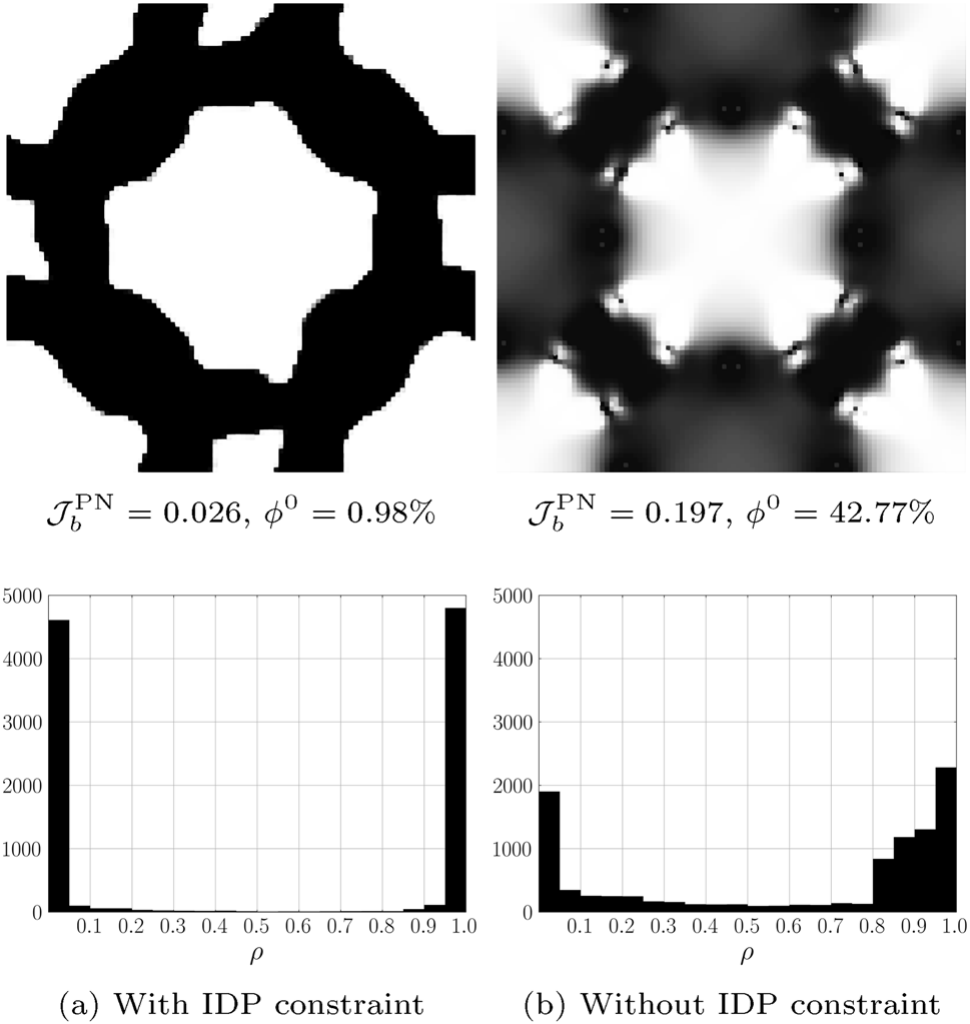
Comparison of geometries generated with (**a**) and without (**b**) the IDP constraint along with the corresponding material distribution histograms (bottom row)

To further demonstrate the benefit of the IDP constraint, a comparative optimization problem is performed with the same settings but without the IDP constraint. The resulting geometry and corresponding material distributions are shown in Figs. 6b and d, respectively. In comparison to the geometry optimized with the IDP constraint, shown in Fig. 6a, a significant amount of intermediate material can be seen, with $\phi ^0 = 42.77\%$ (compared to $\phi ^{0} = 0.98\%$ with the IDP constraint). Furthermore, as discreet structures naturally promote nonlinear behaviour, without the IDP constraint, the lack of discreetness hinders the optimization as evidenced by the large error in the optimized design, ${\mathcal {J}}^{\text {PN}} = 0.197$, compared to ${\mathcal {J}}^{\text {PN}}_b = 0.026$ with the inclusion of the IDP constraint.

### Directional softening

**Fig. 7:**
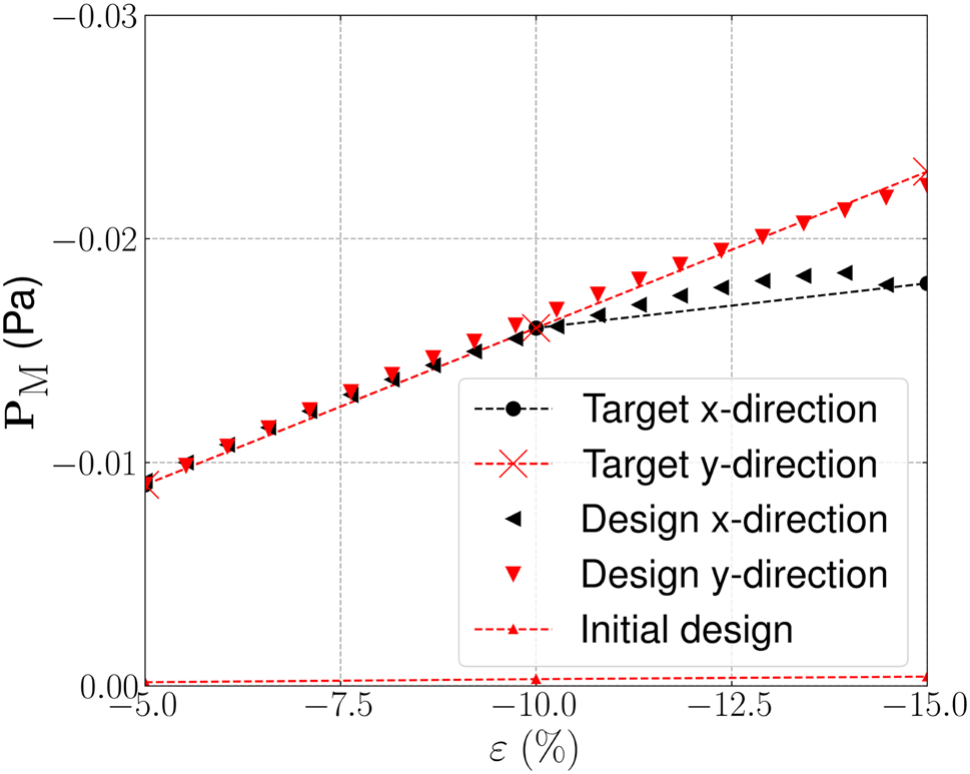
Directional softening—target, initial and optimized finite deformation-based stress–strain relationships in the strain range $\varepsilon \in [-0.05, -0.15]$

**Fig. 8:**
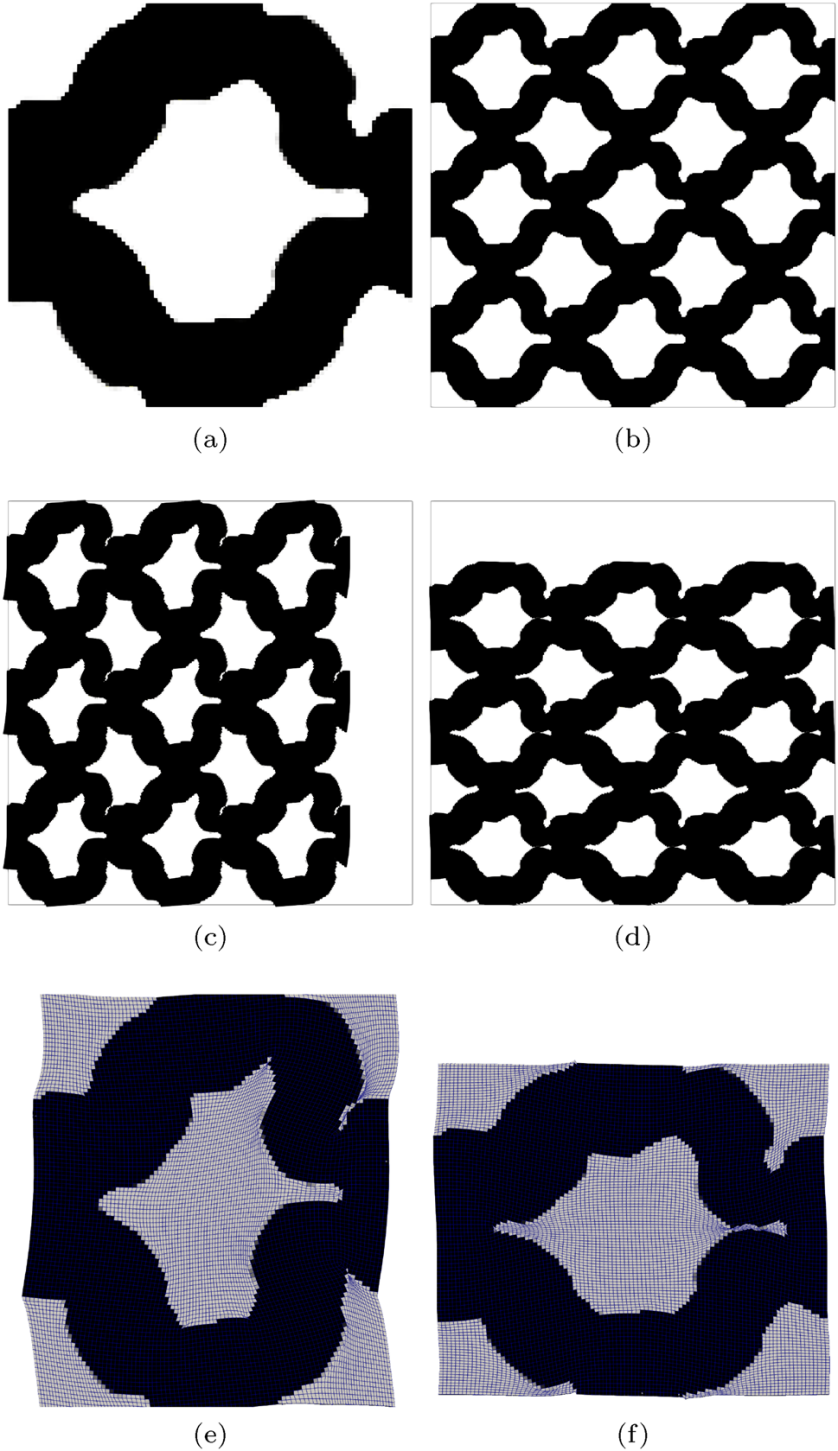
Geometry optimized for directional softening. **a** Unit geometry (${\mathcal {J}}^{\text {PN}}_b = 0.026$, $\phi ^{0}=1.057\%$), **b** array of un-deformed 3$\times$3 unit cells, **c** 3$\times$3 array deformed in x-direction ($\varepsilon _{xx} = -0.15$), **d** 3$\times$3 array deformed in y-direction ($\varepsilon _{yy} = -0.15$). The black box indicates the outline of the un-deformed geometry, **e** close-up of x-deformed unit cell with the FE mesh overlaid, **f** close-up of y-deformed unit cell with the FE mesh overlaid

In the second example directional behaviour is explored. We target a nonlinear stress–strain relationship in the *x*-direction, where again softening is targeted beyond $\varepsilon _{xx} = 0.1$ compressive strain. Independently, in the *y*-direction, a linear relationship is sought, with the stress increasing linearly with strain. The optimizer is again initialised with the material distribution shown in Fig. 3, where for $k=1$, 150 optimization iterations are utilised with 100 iterations utilised for each of the following continuation steps. The targeted and optimized stress–strain relationships are shown in Fig. 7 with the corresponding optimized, un-deformed and deformed geometry, shown in Fig. 8. Good performance is observed in both the *x* and *y*-directions, with ${\mathcal {J}}^{\text {PN}}_b = 0.026$, indicating errors less than 3% between the target and realised stresses. The geometry can be seen to include crisp boundaries negligible amounts of grey material, as $\phi ^0 = 1.06\%$. From the deformed geometry shown in Fig. 8c, it can be seen that the high degree of curvature on the right side of the microstructures induces bending when exposed to compressive strains in the *x*-direction. It should be noted that there appears to be very small regions of near self-contact as this geometry is loaded in the *x* and *y*-direction, as shown in Fig. 8e and f, respectively. To accurately model this behaviour, additional self-contact mechanics should be included, for example as done in Dalklint et al. (2023). This will be the topic of future research. The results shown in Fig. 7 are further validated using smaller void stiffness values. The results of this validation are included in the Appendix 3, where it is found smaller void stiffness values have negligible impact on the structural response of the optimized design.

### Bi-directional stiffening

Next, we present an example where targeted bi-directional stiffening is sought after. Here stiffening behaviour is targeted after beyond 12.5% strain, independently, in the *x* and *y*-directions. The design is optimized by considering three strain configurations $\varepsilon _{xx}, \varepsilon _{yy} = [0.5, 12.5, 20]$. In this example 300 iterations are used for $k=1$ followed by 150 optimization iterations in each of the remaining continuation steps.

**Fig. 9:**
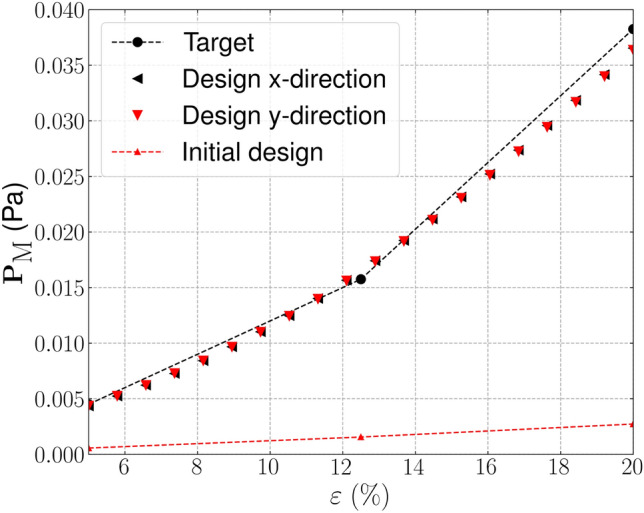
Bi-directional stiffening—target, initial and optimized finite deformation-based stress–strain relationships in the strain range $\varepsilon \in [0.05, 0.18]$

**Fig. 10:**
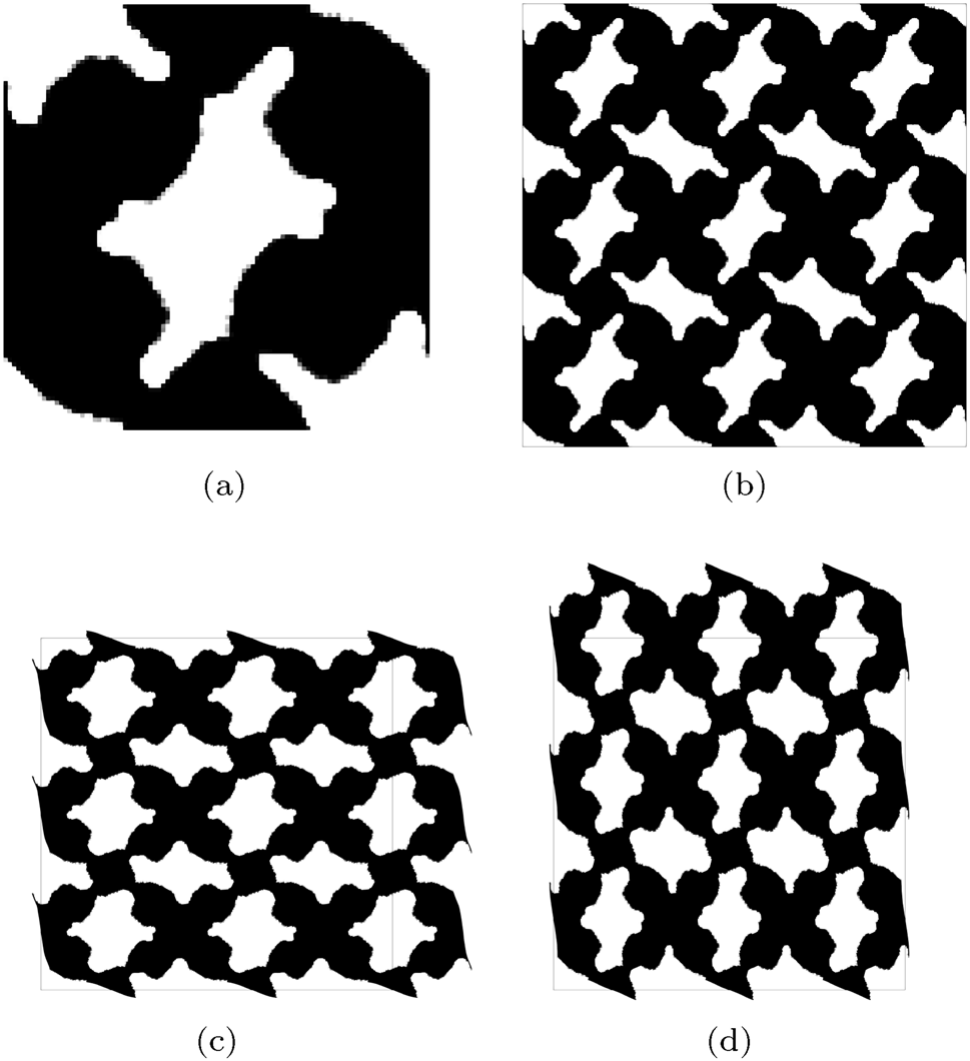
Geometry optimized for bi-directional stiffening. **a** Unit geometry (${\mathcal {J}}^{\text {PN}}_b = 0.046$, $\phi ^{0}=1.364\%$), **b** Array of un-deformed 3$\times$3 unit cells, **c** 3$\times$3 array deformed in x-direction ($\varepsilon _{xx} = 0.18$), d) 3$\times$3 array deformed in y-direction ($\varepsilon _{yy} = 0.18$). The black box indicates the outline of the un-deformed geometry

The target and optimized stress–strain relationships for this problem are shown in Fig. 9, where the optimized design is shown to replicate the targeted stress–strain profile well with an error of ${\mathcal {J}}^{\text {PN}}_b = 0.046$. The corresponding optimized geometry shown in Fig. 10a, contains negligible grey material with $\phi ^{0} = 1.364\%$. From the arrangement of 3$\times$3 unit cells shown in Fig. 10b it can be seen that the stiffening behaviour arises from the chairal-like geometry, that unfolds in the loaded direction to provide additional stiffness, matching the targeted stress–strain profile.

### Directional stiffening

**Fig. 11:**
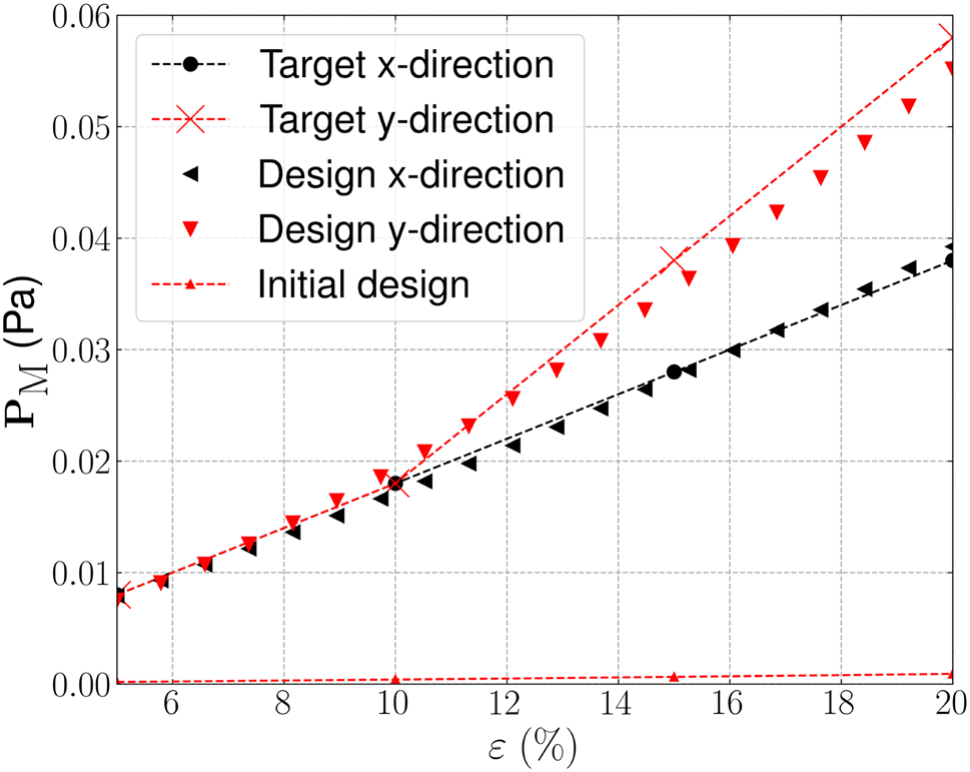
Directional stiffening—target, initial and optimized finite deformation-based stress–strain relationships in the strain range $\varepsilon \in [0.05, 0.2]$

**Fig. 12:**
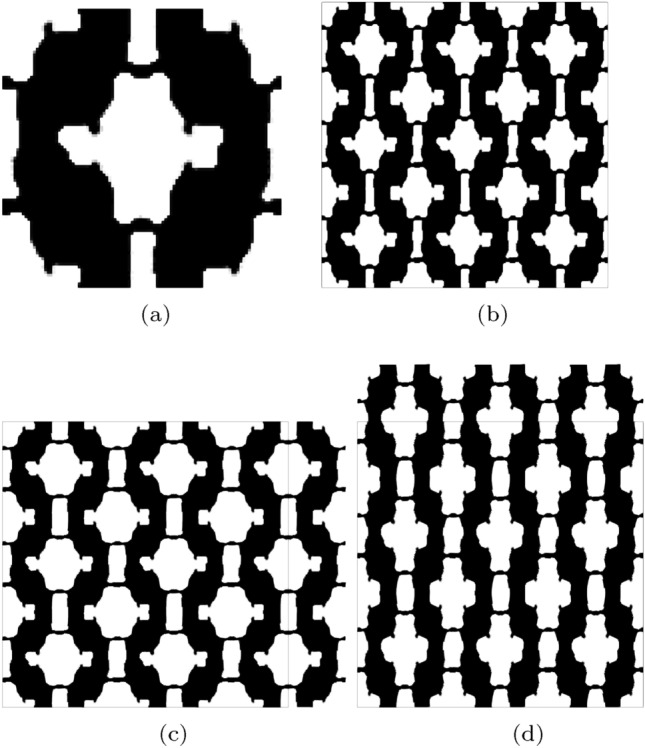
Geometry optimized for directional stiffening. **a** unit geometry (${\mathcal {J}}^{\text {PN}}_b = 0.063$, $\phi ^{0} = 1.49\%$), **b** array of un-deformed 3$\times$3 unit cells, **c** 3$\times$3 array deformed in x-direction ($\varepsilon _{xx} = 0.2$), d) 3$\times$3 array deformed in y-direction ($\varepsilon _{yy} = 0.2$). The black box indicates the outline of the un-deformed geometry

In this last example we target directional stiffening under tension, with a linear behaviour in the *x*-direction and nonlinear stiffening in the *y*-direction, for $\varepsilon _{yy} \ge 0.1$. The stress–strain profile is defined in the finite strain range $\varepsilon _{xx}, \varepsilon _{yy} \in [0.05, 0.2]$ and the optimization is again initialised with the material distribution shown in Fig. 3. The target and optimized stress–strain relationships are shown in Fig. 11. The optimized profile again closely matches the target profile in both directions, with a final error norm of ${\mathcal {J}}^{\text {PN}}_b = 0.063$ with the maximum error between the target and realised stress in the $\varepsilon _{yy} = 0.1$ load case. From the deformed configuration of the optimized geometry shown in Fig. 12d it is clear that the stiffening behaviour in the *y*-direction is induced by material aligning to the load direction, due to the curved sections of material, as the microstructure stretched. In the *x*-direction, thin compliant hinges can be seen which do no cause large increases in stress (stiffness) as the microstructure is stretched in the *x*-direction.

### Effect of initialisation

All the examples shown so far were initialised using the material distribution shown in Fig. 3. As previously explained, it is imperative that periodic inverse design problems are initialised using a heterogeneous material distribution. First, a homogeneous material distribution provides no useful information to the optimizer with regards to achieving nonlinear behaviour; and secondly, a homogeneous material distribution would induce zero microscale fluctuations so no microscale behaviour would be experienced. However, due to the vast design space, with several local minima, the initialisation plays an important role in the performance and topology of the final, optimized design. To demonstrate this, we re-run the previous four problems with different initializations. To generate the initialisation we utilise the Perlin, pseudo-random, noise function, commonly used for procedural terrain generation in computer graphics Perlin (1985). The noise is generated by smoothly interpolating random gradients assigned to each node of the discretized domain, generating a smooth distribution of material.

**Fig. 13:**
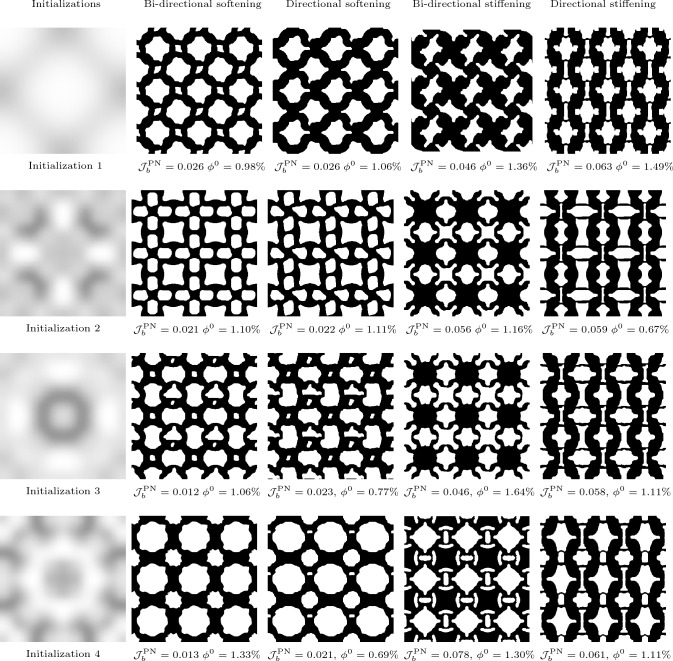
Effect of different material distribution initialisations on the optimized topologies—column **a** Initial material distributions, column **b** Optimized geometries for bi-directional softening, column **c** Optimized geometries for directional softening, column **d** Optimized geometries for bi-directional stiffening, column **e** Optimized geometries for directional stiffening

**Fig. 14:**
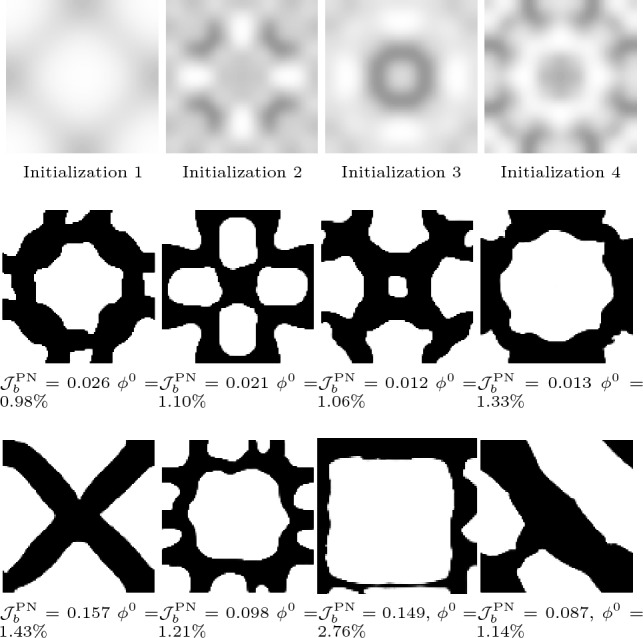
SIMP (*p* = 3) and un-penalized SIMP (*p* = 1) designs—top row) initial material distributions, middle row) optimized geometries for bi-directional softening with *p* = 3, Bottom row) for bi-directional softening with *p* = 1

Using an additional three initializations, we examine a total of four microstructure intializations for each problem. The resulting microstructures are shown in Fig. 13. All initializations result in structures with low errors relative to the prescribed stress–strain curves and contain crisp boundaries, highlighting the robustness of the proposed optimization framework. From these results we observe interesting patterns emerging across the different initializations for each problem. For example, the topologies generated for the directional stiffening case are similar despite different initializations. For each initialization the resulting microstructure contains curved inner boundaries which aligns to the *y*-direction when stretched. By contrast, the bi-directional and directional softening results appear to be be heavily influenced by the initial conditions, suggesting that there are many material distributions that are capable of achieving softening behaviour. This indicates a large feasible design space, where the final local minima that is reached depends on the gradients of the initial material distribution.

## Conclusions

In this work we have presented a framework for designing periodic microstructures with targeted nonlinear stress–strain relationships. Using a deformation-driven homogenization method, homogenized stresses can be easily derived for arbitrary material distributions within a unit cell. Inverse design problems can be tackled using this model whereby targeted nonlinear stress responses are sought after for chosen a set of strain configurations (load cases). In its standard form this optimization problem does not promote discrete designs and as a result hinders the optimization process. To overcome this, we propose the inclusion of an intermediate density penalty constraint where the amount of grey material is directly controlled, encouraging black and white designs with crisp boundaries. We tackle four inverse design problems and demonstrate the ability to systematically design discrete microstructures with directional and bi-directional nonlinear stress–strain relationships. The main conclusions from these results are as follows:

- The proposed framework is able to design microstructures that accurately replicated the targeted stress–strain relationships with less than 10% errors.
- The IDP constraint ensures the formation of discrete microstructure topologies with less than 2% grey material in all cases. As discrete designs better promote nonlinear behaviours, the IDP constraint further aids the optimization convergence.
- While the initial material distribution is found to impact the optimized topologies, the impact is not identical across different problems. Some problems appear to consistently result in similar topologies, suggesting fewer local minima.

This framework lays the foundation for multiscale nonlinear optimization, where nonlinear structures are optimized on the microscale (either concurrently or in a offline manner) to design nonlinear macroscale structures with highly nonlinear structural responses. For example, the design of deployable nonlinear structures which exhibit bi-stable behaviours and or deploy at targeted loads.

Future work should be directed towards the inclusion of self-contact mechanics (c.f. Bluhm et al. (2021); Dalklint et al. (2023)), both for the sake of accurately capturing microstructure behaviour, as well as increasing the range of inverse design problems that may be tackled. In addition, it will be interesting to explore the impact of the IDP constraint when self-contact is included in the inverse homogenization scheme, for example as presented by Dalklint et al. (2023). Furthermore, to improve the manufacturability of the optimized designs, additional constraints should be introduced to avoid the formation of small internal voids or hanging structures. There is ongoing research to perform validation of the optimized structures using both numerical single-scale analysis and through experiments. Lastly, the extension to 3D domains will be considered. The framework presented in this work, directly extends to 3D. However, there are significant computational costs associated with 3D analysis (both in terms of individual FE analyses and the number of strain cases required) which will need to be addressed.

## Figures and Tables

**Fig. 15:**
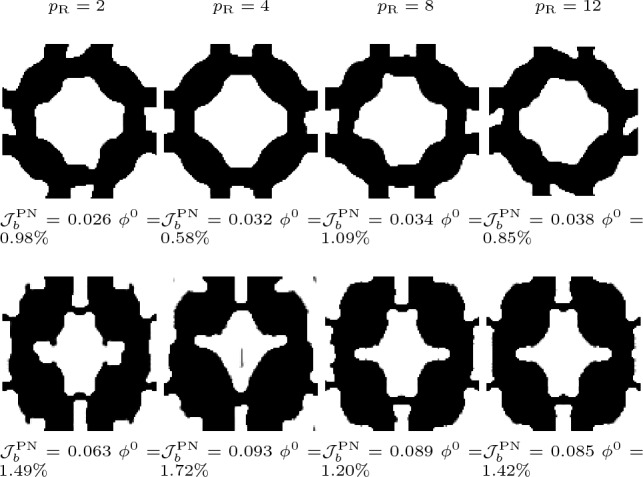
Impact of $p_{\text {R}}$ on the optimized topologies—top row) bi-directional softening, bottom row) directional-stiffening

**Fig. 16:**
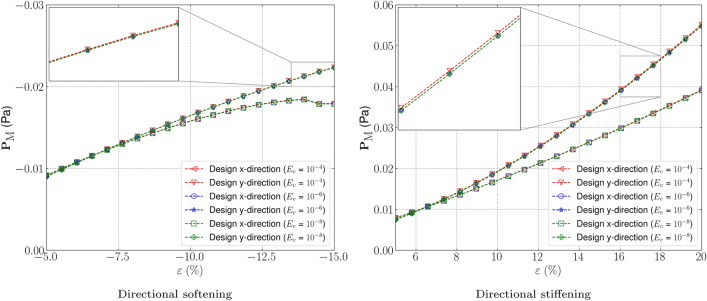
Evaluating the impact of void stiffness values on the structural response of the designs optimized for directional softening (left) and stiffening (right) using $E_{\text {v}} \in [10^{-4}, 10^{-6}, 10^{-8}]$

## Appendix 1: Comparison to un-penalized SIMP

To compare the impact of enforcing the IDP constraint with and without SIMP penalization, the bi-directional softening problem was solved with $p = 1$ using the initializations used in Sect. 5.5. The optimized designs realized using $p = 3$ and $p = 1$ are shown in Fig. 14. The designs optimized using $p = 1$ are found to be significantly worse with errors over 10%.

## Appendix 2: Influence of robust norm parameter

The influence of the choice of norm ($p_\text {R}$) used in Eq. 22 is explored by tackling the bi-directional softening and directional stiffening examples outlined in Sects. 5.1 and 5.4, respectively, using $p_\text {R} \in [2, 4, 8, 12]$. The optimized designs are shown in Fig. 15, where the designs are shown to be almost identical for all four $p_\text {R}$ values in both test cases. However, in all cases the errors are worse when $p_\text {R} > 2$, with the error increasing by 46% and 35% between $p_\text {R}=2$ and $p_\text {R}=12$ for the bi-directional softening and directional stiffening cases, respectively. This is to be expected since increasing $p_\text {R}$ leads to increasingly nonlinear problems resulting it more difficult optimization problems.

## Appendix 3: Influence of void stiffness

To ensure the structural response of the optimized designs are not impacted by the choice of void stiffness value ($E_{\text {v}} = 10^{-4} \;$Pa), we validate the response of the structures optimized for directional softening and stiffening, shown in Figs. 7 and 11, respectively, using smaller void stiffness values ($E_{\text {v}} \in [10^{-4}, 10^{-6}, 10^{-8}]$). The results of this validation are shown in Fig. 16, where it can be seen that smaller void stiffness values have negligible impact on the structural response of the optimized designs.

## Declarations

### Replication of results

The authors wish to withhold the data for commercialization purposes. This includes the inverse homogenization model and a python code implementing the optimization.

### Code availability

The authors wish to withhold the code for commercialization purposes.
